# A new species of genus *Pseudaspidapion* Wanat, 1990 (Coleoptera, Apionidae) from China

**DOI:** 10.3897/zookeys.120.1434

**Published:** 2011-07-25

**Authors:** M. A. Alonso-Zarazaga, Zhiliang Wang, Runzhi Zhang

**Affiliations:** 1 Key Laboratory of Zoological Systematics and Evolution, Institute of Zoology, Chinese Academy of Sciences, Beijing, People’s Republic of China; 2 Depto. de Biodiversidad y Biología Evolutiva, Museo Nacional de Ciencias Naturales (CSIC), José Gutiérrez Abascal, 2, E-28006Madrid, Spain

**Keywords:** Weevils, *Pseudaspidapion botanicum*, *Harpapion*, Beijing, Shaanxi, new species, new combination, morphology, systematics, biology, key

## Abstract

*Pseudaspidapion botanicum* **sp. n.** from China is described and figured. Its host plant is *Grewia biloba* G. Don var. *parviflora* (Bunge) Hand.-Mazz (Malvaceae: Grewioideae). The genus *Harpapion* Voss, 1966 is recorded as new for China and Vietnam and two **comb. n.** are proposed: *Harpapion vietnamense* (Korotyaev, 1985) (from Aspidapion) and *Harpapion coelebs* (Korotyaev, 1987) (from *Pseudaspidapion*). A key to the known species of the genus *Pseudaspidapion* from China is presented.

## Introduction

M. Wanat (1990) erected the genus *Pseudaspidapion* relating it to the genus *Aspidapion* Schilsky, 1901. Alonso-Zarazaga and Lyal (1999) placed it in the tribe Aspidapiini Alonso-Zarazaga, 1990 of the subfamily Apioninae Schoenherr, 1823 (Coleoptera, Curculionoidea). This genus is quite similar to *Aspidapion*, but it can be distinguished from the latter by relatively equal width of elytral striae and interstriae, the presence of fenestrae in the tegminal plate, and the absence of protibial mucrones in males, among other features (Wanat 1990). Apart from the type species, *Apion spadiceum* Wagner, 1908, Wanat also proposed 15 new combinations for species coming from East Africa, India and South China. At present, there are 17 paleotropical species included in the genus. In the Chinese fauna, there were 4 species recorded only from Yunnan province, and these specimens were all collected during the 1950’s (Korotyaev 1985, 1987).

In 2008, we collected a series of specimens of a *Pseudaspidapion*. Further specimens were later found. We consider them to represent a species new to science that we describe below.

In addition to the description of the adult characters, we also provide some biological data for the new species after one year of survey in the Beijing area.

## Material and methods

Materials examined of new species for this study are to be deposited in the Institute of Zoology, Chinese Academy of Sciences, Beijing (IZCAS), the Museo Nacional de Ciencias Naturales, Madrid (MNCN), the Zoological Institute of Russian Academy of Sciences, Moscow (ZIN), the Museum of Natural History, University of Wrocław (MNHW) and the Beijing Botanical Garden, Beijing (BBG).

Type specimens were obtained from ZIN on loan or belong to IZCAS and their data are summarised in Table 1. Information about their condition and taxonomy is given in the Discussion section.

**Table 1.** List of studied type specimens and depositories.

Species	Depository	Examined type material
*Aspidapion inarmatum* Korotyaev, 1985	IZCAS	2 paratypes
*Aspidapion panfilovi* Korotyaev, 1985	IZCAS	Holotype and 1 paratype
*Pseudaspidapion coelebs* (Korotyaev, 1987)	ZIN	1 paratype
*Pseudaspidapion kryzhanovskii* (Korotyaev, 1987)	ZIN	1 paratype
*Pseudaspidapion medvedevi* (Korotyaev, 1985)	ZIN	Holotype
*Pseudaspidapion topali* (Korotyaev, 1985)	ZIN	1 paratype
*Pseudaspidapion vietnamense* (Korotyaev, 1987)	ZIN	Holotype
*Pseudaspidapion yunnanicum* (Korotyaev, 1985)	IZCAS	Holotype and 3 paratypes
*Pseudaspidapion zagulajevi* (Korotyaev, 1987)	ZIN	2 paratypes

Descriptions were made and photographs were taken with a CCD Qimagine MicroPublisher 5.0 RTV mounted on a Zeiss SteREO Discovery V.12. Extended focus images were generated with Auto-Montage Pro 5.03.0061 and edited with Adobe Photoshop CS 5.0 if required. Microscopic slides were studied under a Leica DM 2500 microscope and photos were taken with a Nikon CoolPix 5400. The map was made with the software ArcGIS 9.3. Drawings were made from the original photographs by using the software Adobe Illustrator CS5.0, or directly by using a drawing tube linked to the microscope.

Nomenclature of the rostral parts follows Alonso-Zarazaga (1989) and that of genitalia follows Alonso-Zarazaga (1990).

The dissecting method used follows Alonso-Zarazaga (1990). Abdomens were put into 10% NaOH for several hours until the inner tissues were digested, and the resultant structures were placed on a temporary microscope slide for examination.

After description, the genitalia and other parts of each specimen were placed in DMHF on a plastic card for long term conservation (Steedman 1958; Bameul 1990).

Labels are described as they are (in Chinese), with pinyin romanization or comments in square brackets; labels are separated by semicolons and lines by slashes.

## Taxonomic treatment

### Pseudaspidapion botanicum

Alonso-Zarazaga & Wang sp. n.

urn:lsid:zoobank.org:act:88656E61-918F-4F1B-A3BD-6A3FA714A237

http://species-id.net/wiki/Pseudaspidapion_botanicum

**Figures 1–4.**
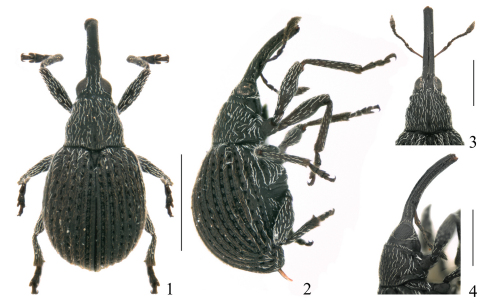
*Pseudaspidapion botanicum* Alonso-Zarazaga & Wang, sp. n. **1** male paratype, dorsal view **2** male paratype, lateral view **3** female paratype, head and rostrum, dorsal view **4** female paratype, head and rostrum, lateral view. Scales: 1–2: 1000 μm; 3–4: 500 μm.

Figs 1 [Fig F2] [Fig F3] 28 

#### Diagnosis.

 This new species resembles *Pseudaspidapion yunnanicum*, but it can be distinguished from the latter by the characters in the table 2.

#### Description

 (male holotype, except where indicated). *Measurements* (in mm): Standard length: 1.82. Rostrum: length: 0.71, maximum width: 0.17. Pronotum: median length: 0.49, maximum width: 0.59. Elytra: median length: 1.38, maximum width: 1.08.

With the general characters of genus *Pseudaspidapion* as described in Wanat (1990).

*Integument*. Generally piceous black (Figs 1–2).

*Vestiture* composed of thick, longer white piliform scales with truncate apex and thin, shorter brownish to greyish acute hairs; scales present on coxae, femora and tibiae, sides of pronotum, meso- and metaventrite, mesoventral process, base of 3rd elytral interstria, metarostrum, and weak subocular patch, sometimes intermediate types present. Antennal scape apex and pedicel with sparse short greyish hairs, club and desmomeres 2–7 covered with dark hairs only. Pronotal vestiture centrifugal, scales both on apex and base perpendicular to the margins, hairs on disc distinctly longer than those on elytra, reaching middle of preceding hair. Elytral vestiture white at base and sides, brownish on disc, interstrial scales large, longer than 2/3 of the interstrial width, in two rather regular rows per interstria, one specialized seta on apical region of 9th interstria.

*Rostrum* cylindrical and moderately robust, in dorsal view 4.18× as long as maximum width, 1.45× as long as pronotum in midline, widest at mesorostrum, pro- and metarostrum with sides almost parallel, metarostrum with two very fine, punctulate dorsal submedial sulci shortened at mesorostral level, and two weak dorsal sublateral sulci, dorsal submedial sulci prolonged on frons, separated, dorsal sublateral sulci prolonged close to ocular margins, metarostrum microreticulate, matt, pro- and mesorostrum smooth, shining, sparsely punctulate; in lateral view weakly and evenly curved, ventral margin forming a weak angle at mesorostrum, each side with a very thin low dorsal sublateral keel running from front margin of eye to upper margin of scrobe and beyond, limiting ventrally the dorsal sublateral sulcus, prorostrum with a marked ventral sublateral sulcus under this keel.

*Head* transverse, frons weakly convex with four rows of relatively deep punctures running from metarostrum and hardly surpassing hind margin of eyes, occiput almost reaching hind eye level, subocular keel reaching middle of eyes, the area between subocular keels microreticulate and impunctate, with a low, fine median keel. Eyes round, moderately convex.

*Antennae* inserted at basal 0.26 of rostral length, scape 5.00× as long as wide, as long as club, 1.18× as long as mesorostral width. Pedicel 2.14× as long as wide, as long as desmomeres 2+3, desmomere 2 1.67×as long as wide, desmomeres 3–5 1.33× as long as wide, desmomere 6 1.14× as long as wide, desmomere 7 1.25× as long as wide, shortly obconical. Club oblong, compact, 2.5× as long as wide, as long as the last 5.5 desmomeres, sutures well marked (Fig 7).

*Pronotum* campaniform, transverse, 0.83× as long as wide, widest just behind middle, constrictions weak, sides weakly dilated at middle, base 1.31× as wide as apex, bisinuate with moderate medial rounded projection towards scutellum, basal flange moderately developed. Prescutellar fovea shallow, as broad as one puncture’s diameter, as long as 2–3 diameters, prolonged in a very fine sulcus reaching middle of pronotum. Discal punctures relatively deep, ca. 0.5–1× diameter apart, interspaces slightly convex, microreticulate.

*Scutellum* large, elongated, triangular, 1.63× as long as wide, with two basal tubercles separated by a weak median depression, in lateral view subacutely prominent, in front view tubercles fused basally; apex constricted and moderately raised, distinctly visible in lateral view.

*Elytra* 1.23× as long as wide, 2.82× as long as pronotum, widest almost at the middle, humeri distinct; striae deep, about as wide as interstriae at base and apex, about half as wide at elytral disc, distinctly catenulate-punctate, punctures round to oblong, space between punctures about 2.00× as long as puncture length, striae apically connected 1+2+9, 3+4, 5+6, 7+8. Interstriae evidently convex with small punctures, surface distinctly wrinkled, not microreticulate, shining. Macropterous (wing of paratype, Fig 8).

*Ventral areas*. Mesocoxae and metacoxae narrowly separated by a distance of 0.26× and 0.38× of their transverse diameter, respectively. Metaventrite ca. as long as mesocoxae. Mesoventral process more prominent than metaventral process. Anterior metaventral rim present, weak. Abdominal ventrites microreticulate, with length ratios along midline: 31-17-6-7-16. Ventrites 1–2 coarsely and densely punctate, 3–4 and base of 5 very sparsely and minutely punctate, apical half of 5 densely punctate with a median convexity. Suture I distinct, distance from hind margin of metacoxae, as long as ventrite 2. Ventrite 5 subsemicircular, transverse, 0.41× as long as wide.

*Pygidium* (from paratype) semicircular, 0.69× as long as wide, apical edge of arc with a flat and glabrous side, punctate medially with interspace microreticulate (Figs 9–10).

**Figures 5–15. F2:**
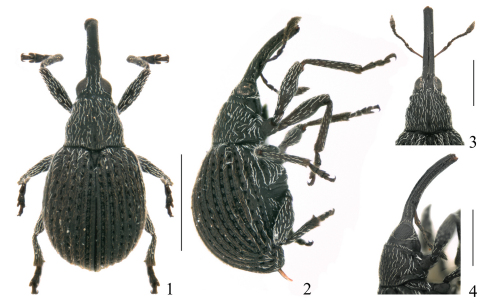
*Pseudaspidapion botanicum* Alonso-Zarazaga & Wang, sp. n., male paratype **5** front leg **6** hind tibia **7** antenna **8** hind wing **9** pygidium, lateral view **10** pygidium, dorsal view **11** tegmen, dorsal view **12** tegmen, lateral view **13** penis, dorsal view **14** penis, lateral view **15** spiculum gastrale. Scales: 5: 500 μm; 6–7, 9–15: 200 μm; 8: 1000 μm.

*Legs*. Metafemora a little more robust than pro- and mesofemora, profemur 2.15× as long as wide, widest at middle, minutely punctate. Protibia almost straight, 6.50× as long as wide (Fig. 5). Protarsomere 1 2.29× as long as wide, tarsomere 2 1.22× as long as wide, tarsomere 3 bilobed, 0.8× as long as wide, lobes narrow, not much dilated outwards, onychium 3.25× as long as wide, projecting from lobes of tarsomere 3 for 0.54× its length. Meso- and metatibiae similarly mucronate, mucro subconical (Fig. 6), about 0.50× as long as tibial apical width. Tarsal claws with conspicuous obtuse basal tooth.

*Genitalia and terminalia* (from topotypic paratypes). Ninth sternite (spiculum gastrale) Y-shaped and slightly winged near arm base, manubrium ca. 2.00× as long as arms (Fig. 15). Penis depressed, moderately curved, pedon with apical plate straight in side view, truncate and slightly constricted apicad in side view; temones about 0.50× as long as pedon; endophallus without large structures (Figs 13–14). Tegminal plate articulated with free ring, laterally developed, enveloping, with parameroid lobes not notched apically, membranous area rhombic in general outline, microsetose apically, reaching middle of parameroid lobes, basal sclerotized area large, each side with 5 short macrochaetae, 2 latero-apical and 3 latero-medial, several sensilla distributed on middle part and posterior margin; fenestrae short, tranverse, narrowly separated; linea arquata visible; prostegium bidentate, apex of projection rounded; median unsclerotized strip moderately elongate, reaching hind margin of fenestrae; manubrium with apex not broadened (Figs 11–12).

**Figures 16–17. F3:**
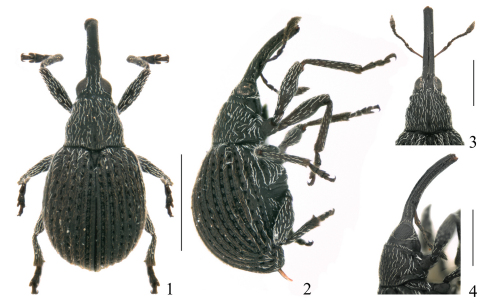
*Pseudaspidapion botanicum* Alonso-Zarazaga & Wang sp. n. **16** female resting on underside of leaf **17** female boring a bud for oviposition, with guarding male.

**Figures 18–33.**
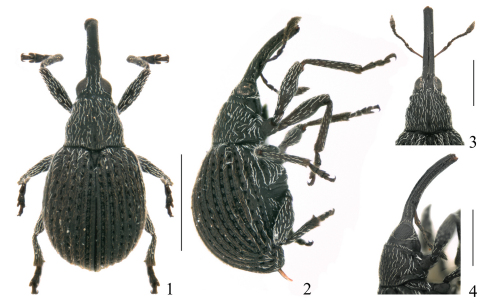
Types of *Pseudaspidapion*, *Aspidapion* and *Harpapion*, in lateral and dorsal views **18–19** *Aspidapion panfilovi* Korotyaev, 1985, female holotype **20–21** *Pseudaspidapion zagulajevi* (Korotyaev, 1987), female paratype **22–23** *Pseudaspidapion kryzhanovskii* (Korotyaev, 1987), female paratype **24–25** *Pseudaspidapion yunnanicum* (Korotyaev, 1985), female holotype **26–27** *Pseudaspidapion topali* (Korotyaev, 1985), male paratype **28–29** *Pseudaspidapion medvedevi* (Korotyaev, 1985), male holotype **30–31** *Harpapion coelebs* (Korotyaev, 1987), male paratype **32–33** *Harpapion vietnamense* (Korotyaev, 1987), male holotype. Scales: 18–33: 1000 μm.

#### Variation.

 **Male paratypes.** Measurements (in mm) (n=10): Standard length: 1.38–2.05 (mean= 1.735). Rostrum: length: 0.52–0.82 (mean= 0.722), maximum width: 0.13–0.19 (mean= 0.164). Pronotum: median length: 0.36–0.53 (mean= 0.479), maximum width: 0.44–0.68 (mean= 0.594). Elytra: median length: 1.06–1.54 (mean= 1.406), maximum width: 0.78–1.18 (mean= 1.038).

*Rostrum* 1.44–1.58× as long as pronotum, 4.00–4.63× as long as wide. *Ventrite* 5 sometimes slightly subtriangular. Otherwise as in holotype.

**Female paratypes** (Figs 3–4). Measurements (in mm) (n=10): Standard length: 1.56–2.05 (mean= 1.831). Rostrum: length: 0.52–0.76 (mean= 0.669), maximum width: 0.130–0.155 (mean= 0.143). Pronotum: median length: 0.37–0.53 (mean= 0.460), maximum width: 0.48–0.65 (mean= 0.571). Elytra: median length: 1.24–1.62 (mean= 1.444), maximum width: 0.88–1.14 (mean= 1.032).

Sexual dimorphism rather weak in this species, females differ from males by a weakly thinner and more evenly curved rostrum with a brighter and less sculptured metarostrum, and all the tibiae without mucros. *Rostrum* 1.35–1.53× as long as pronotum, 4.00–5.07× as long as wide. *Antennae* inserted at ca. basal 0.27 of rostrum. *Ventrite* 5 almost flat, hardly punctured. Otherwise practically as in male.

#### Material examined.

Holotype: ♂: (white, printed): 北京植物园樱桃沟 [Běijīng zhíwùyuán Yīngtáogōu] / 2008.VI.1 / Leg. 王志良 [Wáng Zhìliáng]; (white, printed): IOZ(E)1638667, deposited in IZCAS. This is the Beijing Botanical Garden at Yingtaogou (40°0'51.41"N, 116°12'14.34"E), Haidian district, Beijing (P. R. of China). Paratypes (132♂137♀): 4♂7♀: (white, printed and handwritten): 北京三堡 [Běijīng Sānpù]; (white, printed and handwritten): 1964.VII.20 / leg. 马文珍 [Mă Wénzhēn]; (white, printed): IOZ(E)1638668-1638678; 3♀: (white, printed and handwritten): 北京三堡 [Běijīng Sānpù] / 600m; (white, printed and handwritten): 1964.VIII.21 / leg. 李铁生 [Lí Tiéshēn]; (white, printed): IOZ(E)1638679-1638681; 1♂1♀: (white, printed and handwritten): 北京三堡 [Běijīng, Sānpù] / 1979.VIII.8; (white, handwritten): leg. 廖素柏 [Liào Sùbăi]; (white, printed): IOZ(E)1638682-1638683; 5♂5♀: (white, printed and handwritten): 北京上方山 [Běijīng Shàngfāngshān]; (white, printed and handwritten): 1980.VIII.2 / leg. 廖素柏 [Liào Sùbăi]; (white, printed) IOZ(E)1638684-1638693; 2♂: (white, printed and handwritten): 北京上方山 [Běijīng Shàngfāngshān]; (white, printed and handwritten): 1979.VII.25 / leg. 陈元清 [Chén Yuánqīng]; (white, printed): IOZ(E)1638694-1638695; 2♂2♀: (white, printed and handwritten): 北京居庸关 [Běijīng Jūyōngguān] / 500m; (white, printed and handwritten): 1964.VIII.20 / leg. 李铁生 [Lí Tiéshēn]; (white, printed): IOZ(E)1638696-1638699; 1♂: (white, printed and handwritten): 北京八达岭 [Běijīng Bādálīng] / 700m; (white, printed and handwritten): 1963.VII.25 / leg. 李铁生 [Lí Tiéshēn]; (white, printed): IOZ(E)1638700; 8♂8♀: (white, printed): 北京香山 [Běijīng Xiāngshān]; (white, printed and handwritten): 1963.V.30 / leg. 李铁生 [Lí Tiéshēn]; (white, printed): IOZ(E)1638701-1638716; 1♀: (white, printed and handwritten): 北京香山 [Běijīng Xiāngshān]; (white, printed and handwritten): 1964.V.5 / leg. 马文珍 [Mă Wénzhēn]; (white, printed): IOZ(E)1638717; 1♀: (white, handwritten): 北京香山 [Běijīng Xiāngshān] / 1957.VIII.16; (white, printed): IOZ(E)1638718; 1♂: (white, printed and handwritten): 北京卧佛寺 [Běijīng Wòfósì] / 50m; (white, printed and handwritten): 1962.VI.25 / leg. 王春光 [Wáng Chūnguāng]; (white, printed): IOZ(E)1638719; 1♀: (white, printed and handwritten): 北京卧佛寺 [Běijīng Wòfósì]; (white, printed and handwritten): 1963.IX.3 / leg. 姜胜巧 [Jiāng Shèngqiáo]; (white, printed): IOZ(E)1638720; 1♀: (white, printed and handwritten): 北京卧佛寺 [Běijīng Wòfósì] / 50m; (white, printed and handwritten): 1962.VIII.31 / leg. 谢汝忠 [Xieruzhong]; (white, printed): IOZ(E)1638721; 1♂: (white, printed and handwritten): 北京潭柘寺 [Běijīng Tánzhèsì] / 1975.VII.24; (white, printed and handwritten): leg. 王书永 [Wang Shuyong]; (white, printed): IOZ(E)1638722; 1♀: (white, printed and handwritten): 1964.VII.20 / leg. 马文珍 [Mă Wénzhēn]; (white, printed): IOZ(E)1638723; 2♂3♀: (white, printed and handwritten): 北京圆明园 [Běijīng Yuánmíngyuán] / 1980. VII.7 / leg. 姜胜巧 [Jiāng Shèngqiáo]; (white, printed): IOZ(E)1638724-1638728; 2♂10♀: (white, printed): 北京门头沟军庄西杨坨村 [Běijīng Méntóugōu Jūnzhuāng Xīyángtún] / 2008.VII.27 / leg. 王志良 [Wáng Zhìliáng]; (white, printed): 寄主: 小花扁担木 [Jìzhū: Xiăohuābiăndànmù] / *Grewia biloba* G. Don var. *parviflora* (Bunge) Hand.-Mazz; (white, printed): IOZ(E)1638729-1638740; 5♂8♀: (white, printed): 北京怀柔九渡河镇怀九河 [Běijīng Huáiróu Jiūdùhézhèn Huáijiūhé] / 2008.VI.15 / leg. 王志良 [Wáng Zhìliáng]; (white, printed): IOZ(E)1638741-1638753; 1♀: (white, printed): 北京怀柔三渡河 [Běijīng Huáiróu Sāndùhé] / 2008.V.24 / leg. 王志良 [Wáng Zhìliáng]; (white, printed) IOZ(E)1638754; 46♂24♀: (white, printed): 北京香山植物园樱桃沟 [Běijīng Xiāngshān Zhíwùyuán Yīngtáogōu] / 2008.VIII.19 / leg. 王志良 [Wáng Zhìliáng]; (white, printed): IOZ(E)1638755-1638770, IOZ(E)1638784-1638790, IOZ(E)1638804-1638850; 28♂41♀: (white, printed): 北京植物园樱桃沟 [Běijīng zhíwùyuán Yīngtáogōu] / 寄主：小花扁担木 [Jìzhū: Xiăohuābiăndànmù] / 2008.VI.1 / Leg. 王志良 [Wáng Zhìliáng]; (white, printed): IOZ(E)1638851-1638900, IOZ(E)1638911, IOZ(E)1638928-1638945; 7♂7♀: (white, printed): 北京海淀百望山 [Běijīng Hăidiàn Băiwàngshān] / 2009.VI.21 / leg. 杨干燕 [Yáng Gànyàn]; (white, printed): IOZ(E)1639912-1638918, IOZ(E)16398920-1638926; 2♂1♀: (white, printed and handwritten): 陕西华阴县孟塬 [Shaănxi Huáyīn Mèngyuán] / 450m / 1972.VIII.9; (white, printed): IOZ(E)1639562-1639563, IOZ(E)1639540, deposited in IZCAS.

10♂10♀: (white, printed): 北京香山植物园樱桃沟 [Běijīng Xiāngshān Zhíwùyuán Yīngtáogōu] / 2008.VIII.19 / leg. 王志良 [Wáng Zhìliáng]; (white, printed): IOZ(E)1638781, IOZ(E)1638801, IOZ(E)1638771-1638779, IOZ(E)1638791-1638799, to be deposited in MNCN.

1♂1♀: (white, printed): 北京香山植物园樱桃沟 [Běijīng Xiāngshān Zhíwùyuán Yīngtáogōu] / 2008.VIII.19 / leg. 王志良 [Wáng Zhìliáng]; (white, printed): IOZ(E)1638780, IOZ(E)1638800, to be deposited in BBG.

1♂1♀: (white, printed): 北京香山植物园樱桃沟 [Běijīng Xiāngshān Zhíwùyuán Yīngtáogōu] / 2008.VIII.19 / leg. 王志良 [Wáng Zhìliáng]; (white, printed): IOZ(E)1638783, IOZ(E)1638803, to be deposited in ZINM.

1♂1♀: (white, printed): 北京香山植物园樱桃沟 [Běijīng Xiāngshān Zhíwùyuán Yīngtáogōu] / 2008.VIII.19 / leg. 王志良 [Wáng Zhìliáng]; (white, printed): IOZ(E)1638782, IOZ(E)1638802, to be deposited in MNHW.

The male holotype has not been dissected to avoid any damage, since there are many specimens collected with it.

#### Etymology.

 The new species is named after the first locality where it was found during a collecting visit: the Beijing Botanical Garden. It is a Latin adjective.

#### Distribution.

 The species is known for the moment only from the municipality of Beijing and the province of Shaanxi.

#### Biology.

 *Pseudaspidapion botanicum* sp. n. was collected from *Grewia biloba* G. Don var. *parviflora* (Bunge) Hand.-Mazz (Malvaceae: Grewioideae) (Stevens 2011), which is a common shrub in Beijing and adjacent provinces, blooming from June to July (Tang et al. 1989), this being the season in which the parasite was found on the plant. The adults feed on leaves and flower buds of their host, while they mate and oviposit in the bud. The egg is located in the androecium, and then the larva feeds on the pistil and pupates there.

## Discussion

During this research, we were able to study types of several of the species placed in the genera *Pseudaspidapion* and *Aspidapion* in China in the recent Palaearctic catalogue (Alonso-Zarazaga 2011) (table 1). Until now, females of *Pseudaspidapion coelebs* and *Aspidapion vietnamense* are unknown, and the same can be said of the males of *Pseudaspidapion kryzhanovskii* and *Aspidapion panfilovi*. We found that the holotype (and only known specimen) of *Aspidapion vietnamense*, which has been previously dissected and whose pygidium and tegmen were not conserved, shows several differences from *Aspidapion*, namely, the metatibial mucros are evidently elongate and knicked at their apices (subdentate at the outer margin), the rostrum is clearly dilated at the antennal insertion and distinctly constricted apicad, the setae on the front margin of pronotum are parallel to it, and the apex of the penis is distinctly curved in lateral view, dorsally dentate near the apex. All these characters suggest it is a *Harpapion* Voss, 1966. After a study of the type species of this genus, *Harpapion considerandum* (Fåhraeus, 1871), we consider that, even in the absence of these diagnostic parts, it can be unmistakeably considered a member of this genus and consequently is here transferred: *Harpapion vietnamense* (Korotyaev), comb. n. The same can be said about *Pseudaspidapion coelebs* (Korotyaev, 1987), which show also the characters of the latter genus, and is here formally transferred as well: *Harpapion coelebs* (Korotyaev, 1987), comb. n. This genus will be the subject of a forthcoming paper.

The systematic placement of *Aspidapion inarmatum* is unclear at the moment, but it belongs neither to *Aspidapion* nor to *Pseudaspidapion* or *Harpapion*.

*Aspidapion panfilovi*, known from females until now, possesses several coincident characters with some *Pseudaspidapion*: its more elongate and apparently glabrous elytra, the shallow striae with interstriae about twice as broad as striae on the disc of the elytra, etc. Although it resembles the female of *Pseudaspidapion zagulajevi* very much, we think it is better to postpone any decision on its placement until the male is discovered. This highlights the difficulty of placing in their correct genera the species of Apionidae described only from females, a practice that should be avoided, as that of designating females as name bearing types.

**Figure 34.**
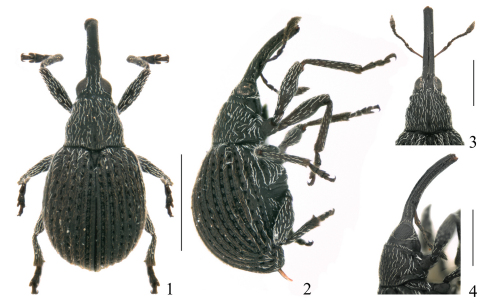
Distribution of the species of *Pseudaspidapion*, *Harpapion* and *Aspidapion* mentioned in the text.

Previously, the genus *Pseudaspidapion* was not known to exceed the Tropic of Cancer. However, the new species *Pseudaspidapion botanicum* reaches 40ºN latitude, an area clearly belonging to the Eastern Palaearctic region (Fig. 34). Thus this genus shows a broader distributional range extending from the Afrotropical across the Oriental to the Palaearctic region. On the other hand, we identified in the collection of IZCAS several specimens of *Aspidapion validum* (Germar, 1817), from Altay prefecture in northern Xinjiang (China). Apart from the species *Aspidapion panfilovi*, whose placement may need a revision as mentioned above, these specimens represent the easternmost distribution record of the otherwise Western Palaearctic genus *Aspidapion*. Therefore, the distribution pattern of *Pseudaspidapion* is mainly Palaeotropical but has expanded to Philippines (Wanat 1990) and to North China while *Aspidapion* is distributed in the Palaearctic region but has also expanded to northeast tropical Africa (Eritrea, Ethiopia). The two genera can still be distinctly separated from each other. Also, we suppose that the blank distributional area of *Pseudaspidapion* between Shaanxi and South China represents a lack of collection records rather than an actual distributional range limit, because the vegetation between these two points includes most of the range of *Grewia biloba* (which includes also Korea) and many other congeneric species of the host genus.

Some of these species are described only from one sex, which makes it difficult to key all of the Chinese species. However, we have tried to provide a key to the known Chinese species (see below). Consequently, more field work is needed to gather specimens of both sexes, and complete our knowledge. We have not studied specimens of *Pseudaspidapion rufopiceum* (Wagner, 1909), a related species, which is included below from the data in its original description.

## Key to the Chinese species of Pseudaspidapion

This key includes also *Aspidapion panfilovi* Korotyaev, 1985 (only known from female), which seems to be related to these species. Body length excludes rostrum.

**Table:** 

1	Meso- and metatibiae mucronate. (males)	2
–	Meso- and metatibiae unarmed. (females)	4
2	Body length 3.1–3.6 mm. Elytral interstrial scales minute, shorter than 1/3 width of interestria, subtransparent, elytra appearing glabrous	*Pseudaspidapion zagulajevi*
–	Body length 1.3-2.2 mm. Elytral interstrial scales larger, longer than 2/3 of the interstrial width, white or dark brown, elytra appearing pubescent	3
3	Body length in general smaller: 1.3–1.6 mm. Body dark reddish-brown, appendages light reddish-brown. Pedicel little longer than wide	*Pseudaspidapion rufopiceum*
–	Body length in general larger: 1.5–2.2 mm. Body and appendages dark brown to piceous black. Pedicel at least 2 × as long as wide	*Pseudaspidapion yunnanicum* / *Pseudaspidapion botanicum* sp. n. (see Table 2 for separation)
4	Body length more than 3.7 mm. Rostrum ca. 2.5 × as long as pronotum. Elytra short, rounded, in side view strongly convex. Scutellum large, base about as wide as interocular distance, triangular, sides straight; in side view completely prominent above elytral outline	*Pseudaspidapion kryzhanovskii*
–	Body length less than 3.7 mm. Elytra oblong-eliptical, in side view moderately convex. Scutellum small, base at most 2/3 as wide as interocular distance, sides concave, forming a basal subpentagonal area prolonged apically in a shaft; in side view, at most some of the tubercles prominent above elytral outline	5
5	Body length more than 2.5 mm. Elytral interstrial scales minute, shorter than 1/3 width of interestria, subtransparent, elytra appearing glabrous	6
–	Body length less than 2.5 mm.Elytral interstrial scales larger, longer than 2/3 width of interstria, white or dark brown, elytra appearing pubescent	3
6	Rostrum ca. 1.8 × as long as pronotum, robust, mesorostrum wider than interocular distance. Basal tubercles of scutellum large, acutely prominent in side view. Body length: 3.1–3.6 mm	*Pseudaspidapion zagulajevi*
–	Rostrum ca. 2.0 × as long as pronotum, fine, mesorostrum at most as wide as interocular distance. Basal tubercles of scutellum small, hardly visible in side view. Body length: 2.7 mm	*Aspidapion panfilovi*

**Table 2.** Comparative diagnostic characters for *Pseudaspidapion botanicum* sp. n. and *Pseudaspidapion yunnanicum*.

	*Pseudaspidapion yunnanicum*	*Pseudaspidapion botanicum* sp. n.
Scales on pronotal disc	Finer, sparser, shorter, tips of posterior scales not or hardly reaching the base of anterior ones	Thicker, denser and longer, tips of posterior scales surpassing the anterior by almost the basal half
Scape	Slender, ca. 0.26 mm long, 7.20–7.60× as long as wide, 1.66–1.85× as long as mesorostral width	Robust, ca. 0.20 mm long, 5.00–5.80× as long as wide, 1.24–1.67× as long as mesorostral width
Rostrum (♀)	Longer, 1.91–2.25× as long as pronotum, thinner, 7.35–10.30× as long as maximum width	Shorter, 1.35–1.53× as long as pronotum, more robust, 4.00–5.07× as long as maximum width
Metarostrum (dorsal view)	Microreticulate	Nearly smooth
Pronotum	Strong subapical constriction and sides moderately rounded behind it	Weaker subapical constriction, sides weakly rounded behind it
Prescutellar fovea	Deep, distinct, lanceolate, not prolonged apicad	Shallow, indistinct, sublinear, prolonged apicad to middle
Scutellum	Two basal tubercles separated medially by a distinctly deep notch, in anterior view tubercles not fused basally	Two basal tubercles separated medially by a weak notch, in anterior view tubercles basally fused

## Supplementary Material

XML Treatment for Pseudaspidapion botanicum
